# Current Management of Traumatic Macular Holes

**DOI:** 10.1155/2017/1748135

**Published:** 2017-01-23

**Authors:** Wu Liu, Andrzej Grzybowski

**Affiliations:** ^1^Beijing Tongren Hospital Eye Center, Capital Medical University, Beijing Ophthalmology and Visual Sciences Key Laboratory, Beijing 100730, China; ^2^Department of Ophthalmology, Poznan City Hospital, Poznan, Poland

## Abstract

Traumatic macular hole (TMH) is not a rare clinical condition, especially in young population. Its prognosis is of complexity and uncertainty, with a relatively high rate of spontaneous closure in some cases. Modern vitrectomy surgery plays an important role in the treatment of TMH, although the functional outcomes may be compromised by the concomitant retinal pathologies. Decision-making about the time of vitrectomy, especially in pediatric patients, remains to be clarified further.

## 1. Introduction

Macular hole is a full-thickness defect of neuroretina in the foveal center, which can cause significant central vision loss. The most common type of macular hole is idiopathic macular holes (IMH), which is caused by the both anteroposterior and tangential vitreous traction on the foveal center [1].

Traumatic macular hole (TMH) is the second most common cause of macular hole. It is defined to macular hole caused by mechanic blunt injury of the eye. Although TMH occurs in 1.4% among closed globe injury cases and to a less extent (0.15%) among open globe injury cases [2], it may sometimes lead to permanent significant vision loss, due to being usually associated with other retinal pathologies, including commotio retinae, diffuse retinal edema, retinal hemorrhage, vitreous hemorrhage, choroidal rupture, photoreceptor and RPE damage, and retinal tears and dialysis.

TMH is more common in younger population, since the young age group is usually associated with sport, recreation, work, and transportation, which is subject to ocular trauma [2, 3]. In a retrospective comparison study with IMH, Huang et al. found that TMH patients were younger (27.11 versus 61.98 y), mainly male (86.3% versus 27.7%), and with worse vision (LogMar VA 1.23 versus 1.06) [3].

## 2. Methodology

The authors performed a literature search through PubMed for reports on traumatic macular hole in human and in English language and also through wanfangdata.com.cn for reports on the same subject in Chinese language. The following key words were used: traumatic macular hole, spontaneous closure, vitrectomy, and optical coherence tomography. Fifty-four articles dating from 1983 to 2016 were chosen for the final analysis.

## 3. Pathogenesis

Although TMH commonly occurs in closed globe injuries caused by blunt ocular trauma, the mechanism of the hole formation remains controversial.

Yokotsuka et al. previously theorized that sudden vitreous separation was the cause of a TMH [4]. Yanagiya et al. noted that most eyes in their 15 cases with a TMH have an attached vitreous; they theorized that it was the force of the impact transmitted to the macula that resulted in rupture of the fovea [5]. In 2001, Johnson et al. proposed contrecoup mechanism of TMH formation [6]. A sudden decrease in the globe's anterior-posterior diameter causes a compensatory equatorial expansion. Such a dynamic change within the volume-fixed globe can lead to horizontal forces and splitting of the retinal layers at the fovea. Yamashita et al. proposed two distinct mechanisms of traumatic macular formation: one that causes immediate visual loss due to primary dehiscence of the fovea and the other that leads to delayed visual loss due to dehiscence of the fovea secondary to persistent vitreofoveal adhesion [7]. Similarly, Hirata and Tanihara proposed that mechanism of the rapid-onset TMH formation was the stretching of the posterior pole as a result of anteroposterior compression of the eyeball [8].

Due to the individual uncertainty of the force imposed on the eye and the inherent structural features of the eye, the extent of the retinal injury and the progression of TMH are still difficult to predict clinically.

## 4. Role of Optical Coherence Tomography

Optical coherence tomography (OCT), especially the latest generation of spectral domain OCT, is the key technique in the management of TMH, which allows detailed assessment of the macular holes parameters, vitreoretinal interface, and other associated macular changes at each presentation. Such detailed observation plays an important role in understanding the pathogenesis of TMH formation [9, 10].

In 1996, Yanagiya et al. observed that most TMHs were elliptical and not round [5]. Huang et al. reported 73 consecutive eyes with TMH with detailed OCT analysis; they proposed 5 different types of TMH according to the presence of cystic edema, retinal detachment, and retinal thinning. Their study demonstrated that the diversity and complexity in clinical presentation are the characteristics of TMH [11]. In another retrospective comparison study with IMH, Huang et al. found that TMHs were more eccentric in shape than the typically circular IMH and had larger basal diameter (1338.45 versus 958.57 mm) and basal area (176.85 versus 77.92 mm2) with a thinner average retinal thickness (248.4 versus 408.8 mm). Visual acuity was negatively correlated with the size of IMHs, but not with any tomographic parameters in TMH. This may be due to the associated damage to the RPE or photoreceptors by the trauma. They found that all cases of TMH showed no posterior vitreous detachment (PVD) and/or opercula by examination and OCT [3]. This is consistent with previous studies that found relatively low rates of PVD in TMH patients [5, 6], which is in opposition to the theory of Yokotsuka et al. that the sudden vitreous separation is the cause of TMH [4].

## 5. Spontaneous Closure

TMHs have been shown to close without any treatment. There are many case reports showing that the spontaneous closures usually took place between 2 weeks and 12 months after the trauma [12–27]. About two-thirds of the reported cases had the holes closure within up to three months of the trauma and nearly all holes closed within six months. Almost all holes are small in size and gain significant visual improvement after the spontaneous closure (Table 1). Karaca et al. reported an unusual horseshoe-shaped traumatic macular tear with spontaneous closure one month after the blunt trauma [26].

There are also some case series documenting the spontaneous closure of TMH, with different closure rate up to 67% [7, 28–33] (Table 2). Mizusawa et al. reported that one (10%) of ten eyes with TMH achieved spontaneous holes closure at a follow-up of 8 months or longer [28]. Li et al. reported that three (10.7%) out of twenty-eight TMHs closed spontaneously after a mean follow-up of 14 months, and the closure occurred within 4.5 months after the injury [30]. Yuan et al. observed that, during a 12-month follow-up, seven (33.3%) of twenty-one cases achieved spontaneous closure of the TMH with significant visual improvement [31]. Chen et al. recently reported twenty-seven eyes with TMH which were followed for at least 6 months and observed spontaneous closure in ten (37.0%) eyes [32]. In a retrospective study of twenty-eight TMH cases, Miller et al. also observed a fairly high spontaneous closure rate (39.3%), in median of 5.6 weeks, with a minimal 1.7 weeks [33]. The largest series by Yamashita et al. reported spontaneous closure in eight (44.4%) of eighteen consecutive cases after a mean follow-up of 8.4 months [7]. The highest closure rate was reported by Tomii et al. that four (67%) of six eyes had spontaneous hole closure during a follow-up of 3 months or longer [29].

Usually, patients with spontaneous closure of TMH shared some common characteristics. First, majority of the patients with spontaneous closure were children or young in age [16, 25]. In Yamashita's large series, all eight patients were young (average age: 14.6 years) and male [7]. Miller et al. also reported that the spontaneous closure rate was greater in children than in adults (50% versus 28.6%) [33]. Second, spontaneous closure mostly occurred in smaller holes [7, 16, 23, 25, 30, 32, 34]. This may implicate that the eye injury is relatively not very severe, or, the hole occurrence is short in course. Third, eyes with spontaneous closure experienced usually absence of PVD [7, 16, 25]. Miller et al. concluded that younger patients without involvement of the posterior hyaloids were likely to have a better prognosis for spontaneous resolution [35].

There were reports of two patients, separately, 55-year-old and 56-year-old, with TMH closed spontaneously [19, 27]. Nasr et al. also reported a case of spontaneous TMH closure in a 50-year-old woman. The authors concluded that the hemorrhagic clot in the TMH base may serve as platelet clumping or a scaffold for glial cell migration and proliferation, thus contributing to spontaneous hole closure [24].

Chen et al. recently explored whether the morphological characteristics on spectral domain OCT can be used to predict spontaneous closure of TMH. In this retrospective study, the authors found that holes with spontaneous closure had smaller minimum diameter (244.9 ± 114.4 versus 523.9 ± 320.0 *μ*m, *p* = 0.007) and less intraretinal cysts (10% versus 76.5%, *p* = 0.001) compared to the holes that did not close spontaneously. Multivariate logistic regression showed that the presence of intraretinal cysts was an independent predictive factor for closure of macular holes, which suggests that the absence of intraretinal cysts on OCT can predict spontaneous closure of TMH. The authors further reviewed that most previously reported cases with spontaneous closure were also not accompanied by intraretinal cysts on OCT [32].

The proposed mechanism of spontaneous closure of TMH encompasses the proliferation of glial cells or retinal pigment epithelial (RPE) cells from bank of the hole's edge to fill the hole bottom and stimulation of astrocyte migration to heal the TMH [36]. Other proposed explanations include formation of a contractile epiretinal membrane resulting in shrinkage and closure of the hole or complete detachment of the posterior hyaloid from the foveal area resulting in the release of an anteroposterior traction [37].

Due to the relatively high rate of spontaneous closure of TMH, it has been suggested that adult patients may be observed for 3 to 6 months after the hole formation, especially in young patients with small holes, good visual acuity, and posterior vitreous adhesion to the hole edges. But surgery may be recommended earlier for pediatric patients to prevent amblyopia, depending on the age of the child [35].

## 6. Vitrectomy

Vitreous surgery for IMH was first reported by Kelly and Wendel in 1991 [38]. Unlike the surgical treatment for IMH, the role of vitrectomy in TMH was less clear, due to the varying contributions of vitreous traction to its pathogenesis and associated retinal damage. However, current surgical techniques are similar to that of the IMH, including removal of the posterior hyaloid, epiretinal membranes, with or without internal limiting membrane (ILM) peeling, and intraocular gas or silicone oil tamponade.

Vitrectomy for TMH has been shown with improved anatomic and visual outcomes in some eyes. A reviewed analysis of surgical outcomes in published reports of vitrectomy for TMH found a successful closure rate of 83% with an overall single operation [35]. Since eyes with TMH are usually associated with different preoperative retinal pathologies, visual outcomes in successfully closed eyes may be unsatisfying. Like most studies, Hou and Jiang reported that 48 (89%) of the 54 eyes with TMH were successfully closed with visual acuity significantly improved after vitrectomy [39], but Yuan et al. did not find significant improvement of visual function in the 18 (69.2%) of 26 cases of TMH that were successfully closed with vitrectomy and gas tamponade [31]. Literature reported outcomes of vitrectomy for cases series of TMH which are summarized in Table 3 [6, 31, 33, 36, 39–50]. This review shows that the anatomic success rate ranges from 45% to 100%, with a median of 92.5%; functional success (2 or more lines' improvement) rate ranges from 27% to 100%, with a median of 84%.

Changes of the hole configuration after vitrectomy were also reported. Rishi et al. reported two TMH cases of delayed and spontaneous conversion of type 2 closure (“flat/open” configuration) to type 1 closure (“flat/closed”) with improved vision months after vitreous surgery [51, 52].

### 6.1. Use of Adjunctive Therapies

Mechanism of macular hole closure treated with vitrectomy involves the stimulation of glial cell proliferation in the hole [53]. To accelerate the healing of the hole, early reports on TMH treatment involved use of adjunctive therapies.

Rubin and colleagues used TGF-*β*2 during vitrectomy for 12 eyes and achieved a closure rate of 67% in eight eyes after the first procedure [40]. García-Arumí et al. obtained a closure rate of 86% in fourteen eyes with full-thickness TMH, with the use of platelet concentrate, after a single surgery [41]. Barreau et al. reported four TMH cases with a mean age of 17 years treated with platelet concentrate and achieved an anatomic successful rates of 75% [42]. Amari et al. and Chow et al. were the first to report successful closure of TMH without the use of adjunctive therapies of TGF-*β*2, platelets, or serum during vitrectomy. Amari et al. achieved a closure rate of 70% in 23 consecutive TMH patients after the first vitrectomy and a rate of 96% after the second intervention. The mean BCVA changed from 20/160 preoperatively to 20/60 postoperatively and 61% of the eyes achieved BCVA of 20/60 or better [44]. Chow et al. reported that fifteen (94%) of sixteen eyes achieved hole closure after vitrectomy and six (38%) had final visual acuity of 0.5 or better [45]. Johnson et al. further studied retrospectively 25 patients who underwent vitrectomy with perfluoropropane (C3F8); twelve of the patients received autologous serum. The macular hole closed in all the 12 (100%) eyes in which serum was used but in 10 (77%) of the 13 eyes in which serum was not used. They found no significant difference in visual acuity outcomes with or without the use of serum [6]. Later, Wachtlin et al. reported a case series of four pediatric patients with TMH treated with vitrectomy with platelet concentrate, ILM peeling, and gas tamponade. Primary closure was achieved by a single intervention in all patients, with visual improvement of three to seven lines after surgery. There were no complications reported [47]. Recently, Hou and Jiang reported their previous series of 54 eyes with TMH that were treated with vitrectomy, platelet concentrate, ILM peeling, and gas tamponade; they found that 48 (89%) of the eyes achieved successful closure with significant visual acuity improvement [39].

### 6.2. Timing of Vitrectomy

In a recent study, eleven eyes with TMH underwent vitrectomy with a median time to intervention of 35.1 weeks. Median time to surgery for the 5 eyes with successful hole closure was 11.0 weeks versus 56.3 weeks for the 6 eyes that failed to close. The authors found no relation between initial OCT dimensions and final hole closure status, and they concluded that surgical intervention was less successful for hole closure when elected after 3 months [33].

### 6.3. Pediatric Vitrectomy

Although TMHs in pediatric patients have a high chance of spontaneous closure, some authors implemented vitrectomy in this subgroup and achieved successful outcomes. Wachtlin et al. reported successful closure of TMH in four (100%) pediatric patients (10–15 years old) after a single vitrectomy with no surgical complications [47]. In another study, four pediatric patients aged under 15 years with TMH following blunt ocular trauma were successfully treated with early vitrectomy. The authors concluded that early vitrectomy seems to be a safe and effective choice in pediatric TMH management, and the risk/benefit ratio of surgery seems to be better than observation [36].

### 6.4. Enzymatic Vitreolysis

Complete removal of the posterior hyaloid is a crucial step for the success of vitrectomy surgery for macular hole [54]. However, it is difficult to mechanically induce complete PVDs in children due to the well-formed vitreous body with strong adhesion between the posterior hyaloid and ILM [55, 56]. Inappropriate maneuvers during PVD induction may result in iatrogenic retinal breaks, inner retina damage, visual field defects, and vitreous hemorrhage. Retinal breaks could trigger an intense proliferative vitreoretinopathy, which will lead to poor anatomic and visual outcomes [56]. For these reasons, enzymatic vitreous liquefaction has been studied to aid PVD induction in pediatric TMH.

Margherio et al. reported “simple and atraumatic” outcomes of 4 pediatric patients with TMH who underwent vitrectomy with 0.4 IU adjuvant of autologous plasmin and 16% C3F8 [43]. Chow et al. reported that, with the use of intraoperative autologous plasmin to facilitate formation of posterior vitreous detachment in ten eyes, fifteen (94%) of sixteen eyes achieved hole closure after vitrectomy and six (38%) had final visual acuity of 0.5 or better [45]. Wu et al. reported a subsequent study of 13 pediatric patients (1–15 years old); after 15 minutes of enzymatic cleavage with 2 IU of autologous plasmin, PVD was noted in 3 eyes and partial PVD in 2 eyes and easily created in the remaining 8 eyes. The macular hole closed in 12 (92%) patients. Among 12 of the 13 patients with VA measurement, 11 (92%) patients had VA improvement of 2 or more lines and six (50%) achieved vision of 20/50 or better; all patients achieved a vision better than 20/200 [48].

With the recent clinical introduction of ocriplasmin (microplasmin/Jetrea; ThromboGenics, Iselin, NJ) which is a recombinant truncated version of plasmin approved by the US Food and Drug Administration for the treatment of symptomatic vitreomacular traction, one would expect a better outcome of enzyme-assisted PVD in pediatric cases of TMH [57].

### 6.5. Intraocular Tamponade

Gas is widely used in repairing any kind of macular holes. It is the surface tension of gas that provides a seal at the site of the hole to prevent reaccumulation of the intraretinal fluid from the vitreous cavity as the hole closes with time. Although gas tamponade is usually recommended over silicone oil for macular hole surgery, silicone oil has also been tried for TMH closure in some cases.

Moura Brasil and Brasil reported a case of a 9-year-old boy with a TMH who was treated with ILM peeling and silicone oil tamponade and who gained vision from 20/300 to 20/70 after silicone oil removal [58]. Ghoraba et al. studied 22 patients with TMH who underwent vitrectomy with silicone oil or gas tamponade. Silicone oil was used in children, patients with large holes, and those with difficulty in strict positioning. With a single surgery, TMH closure was achieved in 67% of cases with silicone oil and 92% with C3F8 [49]. Besides larger size of the holes and unreliability in maintaining a strict postoperative face-down position in child, the lower surface tension of the silicone oil as compared with that of the gas may also be an unfavorable factor for a better surgical outcome in silicone oil tamponaded eyes [59].

### 6.6. Internal Limiting Membrane (ILM)

ILM removal for TMH was studied by Kuhn et al. in a case series of 17 consecutive patients, in whom ILM was removed without any adjuvant therapies. The result showed a 100% closure rate, with vision improving by at least two Snellen lines in 16 (94%) eyes [46]. Ikeda et al. suggested that vitrectomy with ILM peeling is useful not only for TMH closure but also for the release of accompanied severe retinal folds [60]. Wachtlin et al. also performed ILM peeling in treating pediatric patients with success [47].

Recently, inverted ILM flap technique with favorite anatomic and functional outcome for large IMHs was introduced. The inverted ILM flap may act as a scaffold for cell proliferation to promote the closure of the macular hole [61]. Shousha assessed the role of inverted ILM flap as a treatment option for large TMHs. In a prospective noncomparative study of 12 eyes with large TMHs (basal diameter of 1300–2800 um), a 100% closure rate and improvement of best-corrected visual acuity were achieved 6 to 9 months after the surgery [50].

## 7. Conclusion

TMHs are well-known complications of ocular blunt injury. TMHs are relatively rare compared to their idiopathic counterpart, but their visual outcomes and associated injuries can be severe. Besides the mechanism of TMH formation, the decision of whether to operate or simply observe these TMHs is also controversial. Surgical intervention with modern vitrectomy can be very successful in some patients. However, since spontaneous closure of TMH is not very rare, it is reasonable to defer surgical intervention for the first 3 months in amenable patient [33]. No matter whether TMH is spontaneously closed or surgically sealed, the final VA depends less upon the size of the hole than upon the degree of photoreceptor and RPE cell disruption.

Some questions still need to be addressed. What is the key factor(s) for the incidental spontaneous closure of the macular hole? What is the functional consequence(s) of a long period of waiting before vitrectomy? Management guideline of TMH, especially in pediatric patients, needs further to be clarified.

## Figures and Tables

**Table 1:** Literature review of case reports on spontaneous closure of TMH.

References	Patient's age (yrs)	Closure time after injury	Hole size	Initial VA	Final VA
Nunode et al., 1983 [12]	11	15 days	0.2 DD	CF	0.1

Kusaka et al., 1997 [13]	12	4 months	0.1 DD	0.5	1.0
	18	4 months	0.1 DD	0.5	1.0
	19	3 months	0.1 DD	0.2	1.0

Murakami et al., 1998 [14]	12	3 months	0.2 DD	0.03	0.5
	10	3 months	0.1 DD	0.3	0.6

Parmar et al., 1999 [15]	25	15 days	370 *μ*m	CF	0.1

Mitamura et al., 2001 [16]	17	12 months	0.2 DD	0.29	1.0
	13	3 months	0.2 DD	0.2	1.0
	12	3.5 months	0.1 DD	0.28	1.0
	8	4 months	0.2 DD	0.1	1.0
	17	11 months	0.1 DD	0.1	0.8
	13	1 month	0.2 DD	0.2	0.4
	15	3 months	0.1 DD	0.67	1.0

Yamada et al., 2002 [17]	11	18 weeks	0.2 DD	0.2	1.5
	19	4 months	0.2 DD	0.1	0.67
	15	6 months	0.17 DD	0.1	0.67

Yeshurun et al., 2002 [18]	15	5 weeks	600 *μ*m	0.08	0.1

Menchini et al., 2003 [19]	56	2 months	180 *μ*m	0.2	0.67

Carpineto et al., 2005 [20]	10	18 weeks	200 *μ*m	0.1	0.8

Lai et al., 2005 [21]	24	6 weeks	100 *μ*m	0.17	1.0

Chen et al., 2008 [22]	25	2 weeks	n/a	0.01	0.7
		2 weeks	n/a	0.01	0.9

Valmaggia et al., 2009 [23]	9	3 weeks	178 *μ*m	0.04	0.5

Nasr et al., 2011 [24]	50	17 days	n/a	0.03	0.16

de Filippi Sartori et al., 2012 [25]	15	2 months	n/a	0.2	0.8

Karaca et al., 2014 [26]	21	1 month	n/a	CF	CF

Faghihi et al., 2014 [27]	20	2 months	n/a	0.04	0.04
	15	6 months	n/a	0.1	0.4
	25	2 months	n/a	0.06	0.6
	55	1 month	n/a	0.3	0.4

**Table 2:** Literature review of case series reports on spontaneous closure rate of TMH.

References	Number of cases	Mean age (yrs)	Observation time (month)	Closure rate	Closure time after injury (month)
Mizusawa et al., 1996 [28]	10	n/a	8	10%	9
Tomii et al., 1999 [29]	6	n/a	3 or more	67%	2.9 (0.5–5)
Yamashita et al., 2002 [7]	18	n/a	8.4 (4–12)	44%	1.7 (0.27–4)
Li et al., 2008 [30]	28	30	14 (3–131)	10.70%	4.5
Yuan et al., 2015 [31]	21	26	12	33%	1.7
Chen et al., 2015 [32]	27	26	9 (6–37)	38%	n/a
Miller et al., 2015 [33]	28	21	26.4	39%	1.3 (0.4–15.7)

**Table 3:** Literature review of reports on vitrectomy outcomes for TMH.

References	Number of cases	Mean age (yrs)	Adjuvants	Tamponades	Anatomic success^*∗*^	Functional success^#^
Rubin et al., 1995 [40]	12	15	TGF-*β*2	C3F8	67%	67%
García-Arumí et al., 1997 [41]	14	22	Platelet concentrate	SF6	93%	93%
Barreau et al., 1997 [42]	4	17	Platelet concentrate	C3F8	75%	50%
Margherio et al., 1998 [43]	4	13	Autologous plasmin	C3F8	100%	100%
Amari et al., 1999 [44]	23	27	None	SF6	70%	87%
Chow et al., 1999 [45]	16	25	Autologous plasmin	C3F8	94%	69%
Johnson et al., 2001 [6]	25	23	Serum or none	C3F8	96%	84%
Kuhn et al., 2001 [46]	17	26	None	SF6	100%	94%
Wachtlin et al., 2003 [47]	4	13	Platelet concentrate	SF6	100%	100%
Wu et al., 2007 [48]	13	10	Plasmin	C3F8 or sio	92%	92%
Ghoraba et al., 2012 [49]	22	27	None	C3F8 or sio	82%	n/a
Azevedo et al., 2013 [36]	4	12	None	Gas	100%	75%
Hou and Jiang, 2013 [39]	54	27	Platelet concentrate	SF6/C2F6/C3F8	89%	52%
Miller et al., 2015 [33]	11	21	None	Gas or sio	45%	n/a
Yuan et al., 2015 [31]	26	32	None	C3F8	69%	27%
Abou Shousha, 2016 [50]	12	23	None	SF6	100%	n/a

^*∗*^With one operation; ^#^2 lines' visual acuity improvement; sio: silicone oil.
